# A Randomised Controlled Trial of Inhibitory Control Training for Smoking Cessation: Outcomes, Mediators and Methodological Considerations

**DOI:** 10.3389/fpsyg.2021.759270

**Published:** 2021-11-03

**Authors:** Laura K. Hughes, Melissa J. Hayden, Jason Bos, Natalia S. Lawrence, George J. Youssef, Ron Borland, Petra K. Staiger

**Affiliations:** ^1^School of Psychology, Deakin University, Geelong, VIC, Australia; ^2^Cognitive Neuroscience Unit, School of Psychology, Deakin University, Geelong, VIC, Australia; ^3^Centre for Drug Use, Addictive and Anti-social Behaviour Research (CEDAAR), Deakin University, Geelong, VIC, Australia; ^4^Department of Psychology, University of Exeter, Exeter, United Kingdom; ^5^Centre for Adolescent Health, Murdoch Children’s Research Institute, Melbourne, VIC, Australia; ^6^Melbourne Centre for Behaviour Change, School of Psychological Sciences, University of Melbourne, Parkville, VIC, Australia

**Keywords:** smoking, inhibitory control, craving, cognitive training, e-health, devaluation, response inhibition

## Abstract

**Objective:** Inhibitory control training (ICT) has shown promise for improving health behaviours, however, less is known about its mediators of effectiveness. The current paper reports whether ICT reduces smoking-related outcomes such as craving and nicotine dependence, increases motivation to quit and whether reductions in smoking or craving are mediated by response inhibition or a devaluation of smoking stimuli.

**Method:** Adult smokers (minimum 10 cigarettes per day; *N* = 107, *M_*age*_* = 46.15 years, 57 female) were randomly allocated to receive 14 days of smoking-specific ICT (named INST; a go/no-go task where participants were trained to not respond to smoking stimuli) or active control training (participants inhibited responding toward neutral stimuli). Participants were followed up to 3-months post-intervention. This trial was preregistered (Australian and New Zealand Clinical Trials Registry ID: ACTRN12617000252314; URL: https://www.anzctr.org.au/Trial/Registration/TrialReview.aspx?id=370204).

**Results:** There were no significant differences between ICT and active control training groups. Specifically, participants in both groups showed significant reductions in craving, nicotine dependence, motivation and a devaluation (reduced evaluation) of smoking-stimuli up to 3-months follow-up compared to baseline. Inhibition and devaluation of smoking stimuli did not act as mediators. Devaluation of smoking stimuli was an independent predictor of smoking and craving at follow-up.

**Conclusion:** Inhibitory control training (ICT) was no more effective at reducing smoking-related outcomes compared to the active control group, however, significant improvements in craving, dependence indicators and evaluation of smoking stimuli were observed across both groups. A return to basic experimental research may be required to understand the most effective ICT approach to support smoking cessation.

## Introduction

Growing literature has suggested that inhibitory control, which is the ability to inhibit automatic prepotent responses, is impaired in smokers (Smith et al., 2014) and this may contribute to difficulties with quitting. Inhibitory control training (ICT) using the go/no-go task (GNG) has been found to reduce unhealthy food and alcohol consumption more effectively than stop signal tasks (SST) (Allom et al., 2015; Jones et al., 2015). Inhibitory control training using the GNG aims to improve inhibition by training participants to refrain from initiating a response toward salient stimuli (e.g., alcohol or unhealthy food). However, less is known about ICT’s effectiveness for assisting with smoking cessation. Adams et al. (2017) found that one session of lab-based ICT did not lead to greater reductions in smoking at 1-week post-training in smokers who were not focused on quitting compared to a control group. Our research group (Staiger et al., 2018) recently reported on the smoking outcomes of a 2-week online smoking-specific ICT program with nicotine-dependent individuals. No intervention effect was found for cigarette consumption or cessation; however, exploratory analyses provided initial evidence that ICT may assist with smoking reduction for individuals aged under 36 years (Bos et al., 2019).

The current paper reports on the pre-registered secondary outcomes and mediation analyses to complement the above-mentioned primary intervention outcomes (Bos et al., 2019). Although current data indicates that smoking-specific ICT has no significant effect on smoking cessation, it is important to consider that ICT may have improved other important smoking-related outcomes such as craving (Gass et al., 2014). Furthermore, higher nicotine dependence has been shown to be a predictor of failed cessation attempts (Vangeli et al., 2011), and has been associated with poor inhibition (Smith et al., 2014). In contrast, higher motivation to quit is associated with making a quit attempt (Vangeli et al., 2011). To date, no study has assessed the effects of ICT on these important smoking-related outcomes and doing so may clarify whether ICT helps to facilitate smoking cessation.

With respect to mediation, two potential mechanisms have been proposed for ICT. Firstly, that the consistent pairing of target stimuli with not responding (like in GNG-based ICT) results in “learnt” (associative) inhibition (Jones et al., 2015). Indeed, a meta-analysis found that higher percentages of successful inhibitions during ICT resulted in a greater effect size of ICT for reducing alcohol and food consumption (Jones et al., 2015). However, it was not reported whether improvements in individuals’ stimulus-specific inhibition was maintained post-ICT or whether this inhibition acted as a mediator of smoking-specific outcomes. Secondly, another potential mechanism is that ICT devalues (i.e., reduces the positive valence) target stimuli. Evidence for this has been reported in three ICT studies: one food-related (Lawrence et al., 2015), one related to smartphone applications (Johannes et al., 2020) and one targeting smoking (Scholten et al., 2019), although the latter did not report on smoking outcomes. The present study tested both of these potential mechanisms.

For secondary outcomes we hypothesised that, after completing the intervention, smokers who were randomised to the smoking-specific ICT intervention called INST would report significantly less: (1) craving for cigarettes; and (2) nicotine dependence compared to those in the active control group. We also examined two potential mediators of changes in smoking frequency and craving over time: (1) a devaluation of smoking stimuli; and (2) improved response inhibition to smoking stimuli. We also report findings for one pre-registered exploratory hypothesis, which was that smokers who received ICT would report significantly higher levels of motivation to quit smoking compared to smokers in the active control group.

## Method

A detailed protocol for this parallel, two-group, double-blind block randomised controlled trial (RCT; Staiger et al., 2018) and smoking frequency and cessation primary outcomes (Bos et al., 2019) have been recently published. This study received ethics approval (DU-HREC Project Number 2015-298) and was pre-registered (Australian New Zealand Clinical Trials Registry ID: ACTRN12617000252314). Key details are provided below.

### Participants

Eligible participants (see Table 1) were traditional tobacco cigarette (tailored or hand-rolled) smokers (*n* = 107) aged 18 to 60 years (*M* = 46.15, *SD* = 9.38, range = 20–60) who over the past 12-months smoked at least 10 cigarettes per day (*M* = 18.79, *SD* = 6.93, range = 10–44) and met criteria for moderate (*n* = 41) or severe (*n* = 66) Tobacco Use Disorder according to the *Diagnostic and Statistical Manual of Mental Disorders* (5th ed.; DSM-5; American Psychiatric Association, 2013). Participants had: completed at least Year 9 (or equivalent) schooling; a desire to quit smoking; motivation to make a quit attempt during the training stage of the intervention; and regular computer and internet access.

**TABLE 1:** Demographics of participants at baseline.

**Variable**	**Intervention (*n* = 54) M (SD) or %**	**Control (*n* = 53) M(SD) or %**
Age*	46.20 (9.73)	46.09 (9.10)
Gender (% female)*	55.55	50.94
Age commenced smoking*	16.69 (2.41)	15.75 (2.43)
Relationship status (% in a relationship)	74.07	75.47
Country of birth (% Australia)^a^	79.63	88.68
Education (% tertiary educated)*^b^	70.37	62.26
Employed (% yes)*	81.48	79.25
Cigarettes consumed per day*	18.12 (7.12)	19.48 (6.74)
Household with other regular smokers (% yes)	40.74	37.74
**Parents who were regular smokers (%)**		
Both	27.28	33.96
One	55.56	45.28
Neither	16.67	20.75
**In the past 12 months, number of:**		
Quit attempts	1.41 (1.69)	1.81 (3.10)
**Different types of quit aids used (%):**		
None	35.19	20.75
1 type	31.48	45.28
2 types	18.52	28.30
3+ types	14.81	5.66
DSM-5 Tobacco use disorder symptoms*	6.59 (2.11)	6.57 (1.86)
No. ICT/Control training sessions completed*	10.50 (2.91)	10.89 (3.20)

*Published in Bos et al. (2019).

*^a^One person (total) also identified as Australian Aboriginal and/or Torres-Strait Islander.*

*^b^Five people (total) also identified as students.*

Participants were excluded if they primarily used e-cigarettes; had not smoked for 2 weeks or more in the past 3 months; were using psychotropic (e.g., antidepressant, antipsychotic or anxiolytic) or anti-craving medications (e.g., varenicline or bupropion); used nicotine replacement therapy (NRT) during the training phase; engaged in problematic alcohol and/or drug(s) use other than tobacco; or had a history of traumatic or acquired brain injury or a loss of consciousness of over 30 min.

### Go/No-Go Training Tasks

**FIGURE 1:**
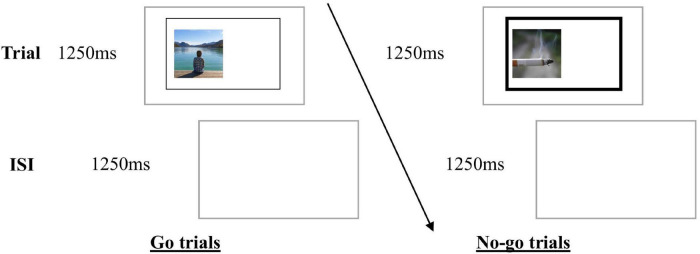
Go/no-go ICT and Active Control Training. Images were presented within a rectangle, followed by an inter-stimulus-interval (ISI). Participants were instructed to indicate as quickly and as accurately as possible the location of an image within the rectangle (left or right) by pressing a computer key (C or M, respectively) when the rectangle was not bolded (go trials). However, when the lines of the rectangle were bold (no-go trials), participants were instructed to refrain from responding.

The online intervention was based on a modified smoking-specific GNG ICT task (therein referred to as ICT; see Figure 1) designed originally by Lawrence et al. (2015). Inhibitory control training consisted of nine smoking-related images (100% no-go) and nine images of relaxing activities such as sitting by a river or lying in a hammock (100% go) and 18 neutral images of clothing (50:50 go/no-go). The control GNG task was identical to ICT except that the stimuli consisted of 18 neutral images only (e.g., household items). For the ICT, images of relaxing activities were chosen for go trials as compared to alternatives for appetitive behaviours like alcohol (i.e., non-alcoholic beverages) or high calorie/high fat foods (e.g., fruit and vegetables) as there are no clear health alternative behaviours for smoking (see Guo, 2018). Neutral images of clothing acted as filler images to increase engagement and difficulty, and to reduce the likelihood of participants identifying the associative patterns within the task (see Lawrence et al., 2015).

Each 10-min computer training session consisted of six blocks of 36 trials (50:50 go/no-go). Each image was displayed once only. At the end of each block, participants were provided with task performance feedback (accuracy and go reaction time) and were encouraged to try to beat their own scores.

### Measures

**FIGURE 2:**
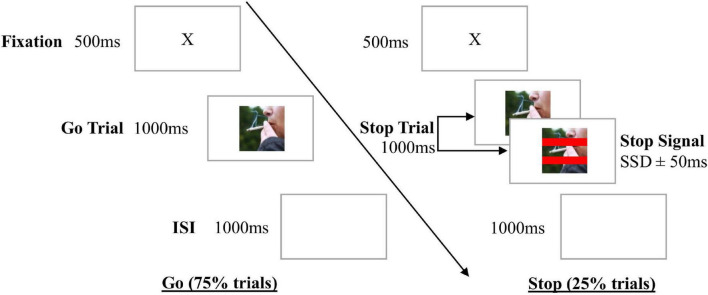
Stop Signal Task (SST). Go stimuli were eight pairs of images of smoking, with one image presenting the cigarette pointing left, and the other its mirror image- with the cigarette pointing right. Each SST begun with a practice block of 10 trials, followed by a test block of 192 trials. After a fixation cross, participants were presented with an image of smoking, followed by an inter-stimulus interval (ISI). Participants were instructed to indicate whether the “lit” or “burnt end” of the cigarette was pointing left or right by pressing a computer key. If red lines (stop signal) appeared across the image, participants were instructed to not respond (stop trials, 25% trials). The stop signal appeared at a short delay (stop signal delay or SSD) after the go stimulus, which began at 250 ms on the first stop trial, and then adjusted by 50 ms in a staircase manner (increased after successful stop trials, or decreased after failed stop trials) so participants had approximately 50% accuracy and converged on a mean SSD.

The following psychometrically reliable and valid measures were used: smoking-related stop signal task (SST; Logan et al., 1997), Fagerström Test of Nicotine Dependence (FTND; Heatherton et al., 1991), Timeline Follow-Back (TLFB; Robinson et al., 2014), and visual analog scales for craving, motivation and the stimulus evaluation test (also see Staiger et al., 2018). Stop signal reaction time (SSRT), a measure of response inhibition from the SST (see Figure 2) was calculated using the mean method (Logan et al., 1997). Data of participants who exhibited accuracy outside of 40%–60% on stop trials and/or less than 70% on go trials during the SST was excluded [based on Congdon et al. (2012)]. The FTND was used as a more phasic measure as our interest was in changes over time.^1^ Smoking frequency measured using the TLFB was defined as mean number of cigarettes consumed per day. The stimulus evaluation test (adapted from Lawrence et al., 2015) involved participants rating the valence of each image of smoking and relaxing activities from the ICT intervention on a 100 mm visual analog scale. Craving for cigarettes and motivation to quit smoking were also measured using visual analog scales. For the abovementioned measures, higher scores indicated higher severity of nicotine dependence or frequency of smoking, poorer inhibition or stronger behavior (e.g., more motivation or more strongly valued visual stimuli). Time (in hours) since last cigarette smoked prior to each training (ICT or active control) session was also collected.

### Procedure

Smokers were screened for eligibility via phone or online survey. Eligible participants were instructed to abstain from smoking for 2 h prior to meeting with a researcher at the university, where they provided informed consent, completed the baseline assessment (TLFB, questionnaires, SST) and were reminded that they were required to make a quit or cessation attempt during the 2-week training period. Participants were told the aim of the study was to “investigate which of the two tasks was more effective” to minimise unblinding. They were then randomised by the computer program’s inbuilt algorithm, and completed their first ICT or control GNG training session with the researcher present to ensure they understood the task.

Following session one, participants were emailed a web link to access the online training and instructed to complete this training once per day for the next 13 days and in a quiet place whilst craving cigarettes. More frequent use or making up for missed sessions was not enabled within the program. SMS reminders to complete training were sent twice per week during the training period.

At all three follow-ups (post-intervention, 1-month and 3-months), participants completed the TLFB via phone with a researcher (naïve to group allocation), then completed the online questionnaires and the SST. After the completion of each follow-up participants received a $20 gift card.

### Analytic Strategy

Any methods not reported in this brief report are detailed in the Supplementary Material. Multiple Imputation by Chained Equations (MICE; Azur et al., 2011) was used to address missing data. Analyses covaried for age due to a potential age effect (Bos et al., 2019) on the outcomes.

#### Secondary Outcomes

Analyses of secondary outcomes used mixed effects linear regression models with random intercept (to account for clustering of time points within individuals) in Stata 15 (StataCorp, 2017). Specifically, we regressed each outcome on to a variable denoting timepoint (i.e., baseline vs. post-intervention vs. 1-month follow-up vs. 3-month follow-up; note that only baseline and post-intervention available for SST), group (i.e., intervention vs. control), and a timepoint × group interaction. Effect size was measured using Cohen’s *d*_*z*_ for paired data and Cohen’s *d* for between group effects. This analysis was then repeated after removing smokers who had abstained at any time after the training period (n_total removed_ = 6).

#### Mediation Analyses

Mediation analyses were conducted in Mplus Version 8 (Muthén and Muthén, 2017) using four time point autoregressive longitudinal mediation models (MacKinnon, 2008). Briefly, this involved estimating autoregressive and cross lagged paths between the outcomes (e.g., smoking) and mediation effects over time. We also explored whether (1) evaluation of smoking stimuli and (2) inhibition had direct effects on smoking frequency and craving, independent of group status using mixed effects multiple regression models.

## Results

The final intent-to-treat sample was 107 (two participants withdrew, and one was removed for using NRT during the training period [see Bos et al. (2019) for details]. Compliance for smoking no less than 2 h prior to each of the fourteen ICT or control training sessions was 60.64%, with 39.36% smoking less than 2 h before training and 35.30% smoking between 2 and 3 h beforehand. There were no significant differences between groups (ICT vs. active control) in demographics and secondary outcomes at baseline, and no main effects of group for any secondary outcomes (any results not provided within this report were detailed in the Supplementary Material). There were no group by timepoint interactions in predicting secondary outcomes; however, we found a significant main effect of timepoint on craving, *F*(3, 1009.7) = 8.56, *p* = ≤ 0.001; nicotine dependence, *F*(3, 1096.5) = 21.69, *p* < 0.001; motivation to quit *F*(3, 1166.1) = 13.14, *p* < 0.001; and evaluation of smoking stimuli, *F*(3, 1457.3) = 13.43, *p* < 0.001 (see Table 2). Specifically, both groups showed reductions in craving, nicotine dependence and evaluation of smoking stimuli at all follow-up time points compared to baseline; with mostly moderate to large effect sizes (*d*_*z*_ from −0.31 to −0.91). Reductions in motivation to quit smoking showed small to moderate effect sizes (*d*_*z*_ from −0.27 to −0.67).

**TABLE 2:** Estimated marginal means (EMM) and standard errors (SE) of each secondary outcome by group over time.

		**Baseline**	**Post-intervention**		**1-Month follow-up**		**3-Month follow-up**	
	**Group × Time interaction on outcome**	**EMM (SE)**	**EMM (SE)**	***d_*z*_* [95% CI]**	**EMM (SE)**	***d*_*z*_ [95% CI]**	**EMM (SE)**	***d*_*z*_ [95% CI]**
Craving	*F*(3, 1440.5) = 0.58, *p* = 0.63							
Intervention		46.44 (3.71)	32.03 (3.97)	−0.44** [−0.72, −0.16]	32.03 (4.04)	−0.45** [−0.73, −0.17]	34.54 (4.71)	−0.31* [−0.58, −0.03]
Control		47.99 (3.68)	31.87 (4.24)	−0.48** [−0.76, −0.19]	35.67 (4.39)	−0.35* [−0.63, −0.08]	29.71 (5.00)	−0.47** [−0.75, −0.18]
FTND	*F*(3, 1392.6) = 0.98, *p* = 0.40							
Intervention		5.41 (0.30)	3.80 (0.32)	−0.78*** [−1.08, −0.47]	3.94 (.35)	−0.64*** [−0.93, −0.34]	3.75 (0.35)	−0.72*** [−1.02, −0.42]
Control		5.72 (0.31)	3.79 (0.33)	−0.91*** [−1.22, −0.58]	4.57 (0.35)	−0.50*** [−0.78, −0.21]	4.43 (0.40)	−0.48** [−0.76, −0.19]
**SST**								
SSRT	*F*(1, 578.0) = 0.01, *p* = 0.92							
Intervention		264.70 (8.82)	264.72 (10.64)	0 [−0.27, 0.27]	−		−	
Control		258.61 (9.33)	256.84 (12.66)	−0.02 [−0.29, 0.25]	−		−	
Go RT	*F*(1, 452.1) = ≤ 0.01, *p* = 0.97							
Intervention		662.68 (13.63)	652.52 (19.25)	−0.07 [−0.33, 0.20]	−		−	
Control		661.82 (13.82)	652.52 (16.69)	−0.07 [−0.34, 0.20]	−		−	
**Evaluation of Images**								
Smoking	*F*(3, 1638.0) = 0.30, *p* = 0.83							
Intervention		51.53 (3.26)	34.32 (3.74)	−0.60*** [−0.89, −0.31]	36.20 (3.75)	−0.53*** [−0.82, −0.25]	33.83 (3.86)	−0.60*** [−0.89, −0.31]
Control		51.76 (3.30)	37.15 (3.86)	−0.50*** [−0.78, −0.21]	41.89 (3.99)	−0.33* [−0.60, −0.05]	36.97 (4.05)	−0.48*** [−0.77, −0.20]
Relaxing activities	*F*(3, 2435.6) = 0.98, *p* = 0.40							
Intervention		72.68 (2.31)	75.43 (2.56)	0.16 [−0.11, 0.42]	73.21 (2.61)	0.03 [−0.24, 0.30]	72.84 (2.50)	0.01 [−0.26, 0.28]
Control		79.04 (2.33)	76.34 (2.64)	−0.15 [−0.42, 0.12]	78.15 (2.80)	−0.05 [−0.32, 0.22]	78.50 (2.61)	−0.03 [−0.30, 0.24]
Motivation	*F*(3,1196.5) = 0.72, *p* = 0.54							
Intervention		79.25 (3.93)	70.77 (4.29)	−0.26 [−0.53, 0.01]	65.04 (4.58)	−0.42** [−0.69, −0.14]	61.29 (5.26)	−0.46** [−0.74, −0.18]
Control		82.80 (3.97)	74.03 (4.37)	−0.27*^a^ [−0.54, 0.01]	70.12 (4.60)	−0.37** [−0.65, −0.09]	56.94 (5.20)	−0.67*** [−0.97, −0.37]

*The presented within groups *d*_*z*_ is that specific time point compared to baseline. Negative *d*_*z*_ and smaller EMM than baseline denote an improvement in secondary outcomes. EMM = Estimated Marginal Means; SE = Standard Error; CI = confidence interval. FTND = Fagerström Test of Nicotine Dependence, SST = stop signal task, SSRT = stop signal reaction time (measure of response inhibition), Go RT = reaction time on go trials. All analyses were adjusted for age.*

*p < 0.05; **p < 0.01; ***p < 0.001. *^a^*p* = 0.049.

Analyses repeated with abstinent participants removed (*n*_intervention_ = 49; *n*_control_ = 52) showed that reductions in craving, nicotine dependence, evaluation of smoking stimuli and motivation to quit were not different to the main analyses using the full dataset. There were no changes in smoking-specific inhibition across time points observed in either group.

Analysis of the longitudinal autoregressive mediation models showed no evidence to suggest that changes in smoking frequency or craving were mediated by changes in inhibition or evaluation of smoking stimuli (*p* = 0.44 to 0.99 for paths of interest: see Supplementary Figures 1–4 in Supplementary Material for further details). Mixed effects regression models revealed that the change in evaluation of smoking stimuli between baseline and post-intervention significantly predicted smoking and craving at all follow-ups, independent of group (see Supplementary Material). Changes in response inhibition did not independently predict smoking and craving at follow-ups. Additional exploratory analyses found that neither age nor the change in motivation to quit over the training period acted as moderators (three-way interaction with time and condition) or independent predictors (interaction with time) for improvements in craving and nicotine dependence (see Supplementary Material for detailed method and results).

## Discussion

This paper reported on the RCT outcomes of online smoking-specific ICT for heavy dependent smokers: craving, nicotine dependence and motivation to quit. Additionally this paper examined whether (1) evaluation of smoking stimuli and/or (2) inhibition acted as mediators between groups and (a) smoking or (b) craving. ICT was no more effective than the active control group for improving smoking-related outcomes, and both groups showed significant reductions in craving, nicotine dependence, and devaluation of smoking stimuli at all follow-ups compared to baseline. Furthermore, both groups reported *reduced* motivation to quit at 1-month and 3-month follow-ups – opposite to what might have been expected from the decline in smoking-related outcomes. Importantly, inhibition and smoking devaluation did not act as mediators between ICT and observed reductions in smoking or craving, with devaluation instead acting as an independent predictor of reductions in smoking and craving across all follow-ups. This suggests that devaluation in smoking may not be driven by ICT.

While no effect of group was found, the current study observed overall small-moderate significant reductions in craving and moderate-large significant reductions in nicotine dependence across all follow up time points. These effects occurred alongside an overall significant reduction in cigarette consumption, as reported in Bos et al. (2019) and were still present with abstainers removed. While findings from the present study were contrary to the hypotheses, failure to detect differences in craving or dependence between groups may be due to general inhibition training effects from using an active control task and/or self-monitoring of cigarette use (see Bos et al. (2019) for discussion). Significant reductions in craving and nicotine dependence in the absence of abstainers suggests that findings were not solely driven by those who had quit. It is also important to note that sustained reductions in cigarette consumption has also been observed in the absence of quit attempts (Yong et al., 2012). However, a number of other important methodological issues warrant discussion as they have important implications for how we might interpret these non-significant findings.

It is important to consider the potential influence that nicotine satiation may have on the measurement of inhibition. Charles-Walsh et al. (2014) found that smokers at 3-h abstinence did not display deficits in response inhibition, and Tsaur et al. (2015) suggested that deficits may not appear until as late as 72 h nicotine abstinence. Whereas, Grabski et al. (2016) found that smokers who were abstinent for at least 10 h displayed these deficits in inhibition. These findings align with evidence that nicotine improves inhibition (as measured by the SST) in healthy non-smokers (Logemann et al., 2014). Additionally, healthy controls have displayed increased activation in prefrontal regions (measured using fMRI) during successful inhibition on stop trials of the SST after nicotine administration (Kasparbauer et al., 2019). Taken together, this suggests that the neurochemical effects of nicotine may improve inhibition and potentially mask inhibitory deficits, which do not appear until at least 10 h post cigarette consumption. In the current study, the majority of participants smoked 3 h or less prior to each training session. It is possible that when satiated, nicotine may have nullified potential deficits in inhibition, making efforts to improve inhibition redundant. This is a potentially serious limitation in the effectiveness of ICT with smokers unless they have been abstinent for a few days. This demonstrates the critical need to return to laboratory style studies to investigate and understand the relationship between nicotine satiation and inhibition.

Another important consideration specifically relates to the measurement of inhibition. In the current study, training used the GNG whereas inhibition was measured using the SST. Whilst both are measures of inhibition, the SST arguably measures top-down inhibition or action cancellation, whereas the GNG is thought to measure automatic bottom-up inhibition or action restraint (Verbruggen and Logan, 2008; Swick et al., 2011; Littman and Takács, 2017). Interestingly, Jones et al. (2018) also found no change in alcohol-specific inhibition following ICT when measured using an alcohol-specific SST. The use of SST to assess inhibition and the effectiveness of GNG ICT may be problematic as the two tasks are thought to assess different aspects of inhibition. Future ICT research should therefore consider employing measures of automatic inhibition, such as slowed response latency to respond to formerly no-go associated stimuli (e.g., Best et al., 2016).

Both groups (ICT and active control) showed significant reductions in the positive evaluation of smoking cues at all follow-ups when compared to baseline. This is similar to findings by Scholten et al. (2019), where smokers showed a devaluation of smoking cues immediately after one ICT session, however, no follow-ups were conducted. Findings of the current study builds upon this by showing that devaluation of smoking cues was maintained long after their last training session, and predicted reductions in smoking frequency and craving. Contrary to study predictions, this effect was also observed in the active control group (not exposed to smoking images) and not just the ICT group. Future studies are needed to clarify whether this may have occurred due to non-specific trial effects (which may have reduced smoking or craving) on devaluation, or a reduction in reactivity to smoking cues alongside cigarette cessation or reduction (Balter et al., 2015). Studies could also consider an active control condition where participants are exposed to smoking images without ICT. Despite the limitations, the observed devaluation of smoking stimuli and its effects on smoking and craving is encouraging and warrants further consideration in future studies.

Other aspects of the study design require consideration. The sample size was powered for the primary outcomes in the expectation of moderate effect sizes (which may have been optimistic), and underpowered for detecting small mediation effects. This limits the interpretation of the non-significant mediation effects on smoking or craving. However, this study was strong in that cigarette consumption was self-report via face-to-face and phone interviews using detailed time line follow-back, which increased accuracy. Although these data collection methods may have been affected by social desirability bias (Latkin et al., 2017; Zhang et al., 2017), there is some suggestion that collecting information regarding consumption (Yeager and Krosnick, 2010) and quit attempts (Persoskie and Nelson, 2013) from smokers is not affected by such bias, and we see no basis to expect differential bias between groups. The observed drop in nicotine dependence post-training, which includes overall estimates of consumption (collected via online survey), was consistent with detailed reported cigarette consumption. This increases our confidence that consumption was measured appropriately, although a biochemical measure for verifying cigarette consumption was not used (Connor Gorber et al., 2009). Future studies should consider including biochemical verification methods of tobacco use, e.g., cotinine, to confirm self-reported cigarette consumption.

An RCT examining alcohol-specific ICT has also reported non-significant findings (Jones et al., 2018). It is possible that unlike the success of ICT for food intake, smoking and alcohol consumption may not be impacted by ICT. Alternatively, further research into intervention design is needed before any conclusions about ICT for reducing smoking and alcohol consumption can be drawn (e.g., types of stimuli for both intervention and control conditions, number of sessions and tailored stimuli type [Staiger and White, 1991)]. It has recently been suggested that the selection of images used as healthy stimuli in contrast to the target behaviour may be important, with Manning et al. (2021) reporting that recipients of cognitive bias modification showed increased approach bias toward non-alcoholic beverages concurrently with increased avoidance bias toward alcohol. Using images of relaxing activities (the current study) and neutral stimuli [e.g., stationary in Adams et al. (2017)] for opposing images to cigarettes have both produced non-significant findings. Future experimental studies could consider trialing various alternative images to cigarettes (e.g., nicotine replacement therapies) to see if these improve the effectiveness of ICT for smoking cessation.

In conclusion, results of the current study suggested that there is no benefit of smoking-specific ICT compared to an active control group, as both groups showed improved craving and nicotine dependence at all follow-ups, and reduced motivation to quit at 1-month and 3-months post-intervention. In addition, no evidence of inhibition or devaluation of smoking stimuli acting as mediators was found, with stimulus devaluation instead independently predicting improvements in smoking and craving. Potentially methodological issues such as non-specific trial effects, nicotine satiation and choice of inhibition measure may have contributed to the reported findings. Therefore, future studies should consider employing an experimental design and addressing these methodological issues.

## Data Availability Statement

The datasets presented in this article are not readily available because the authors do not have ethics approval to make the dataset public. Requests to access the datasets should be directed to PS, petra.staiger@deakin.edu.au.

## Ethics Statement

The studies involving human participants were reviewed and approved by Deakin University Human Research Ethics Committee (DUHREC), Deakin University, Geelong, Australia. The patients/participants provided their written informed consent to participate in this study.

## Author Contributions

LH was responsible for the conceptualisation of the secondary outcomes and mediator variables, data analysis, and preparation of the first draft of the manuscript. PS, MH, and NL conceptualised the study. NL also contributing the model for ICT and active control task development. GY contributed statistical analysis expertise. RB provided input for smoking measures and data interpretation. LH and JB were involved in data collection. PS provided senior oversight of the project. All authors commented on the final manuscript.

## Conflict of Interest

The authors declare that the research was conducted in the absence of any commercial or financial relationships that could be construed as a potential conflict of interest.

## Publisher’s Note

All claims expressed in this article are solely those of the authors and do not necessarily represent those of their affiliated organizations, or those of the publisher, the editors and the reviewers. Any product that may be evaluated in this article, or claim that may be made by its manufacturer, is not guaranteed or endorsed by the publisher.

## Footnotes

1 Participants with 100% days abstinent at a follow-up period (Bos et al., 2019) did not complete the FTND (n_total_ = 3 at 1 month, n_total_ = 5 at 3 months post-intervention), and were deemed to have a FTND total score of 0.
