# Supplementary material for: A Randomised Controlled Trial of Inhibitory Control Training for Smoking Cessation: Outcomes, Mediators and Methodological Considerations
Source: Front Psychol. 2021 Nov 3;12:759270. doi: 10.3389/fpsyg.2021.759270 (PMC8595834; doi:10.3389/fpsyg.2021.759270)
Supplement: Supplementary file 1 [file Data_Sheet_1.pdf]

A Randomised Controlled Trial of Inhibitory Control Training for Smoking Cessation: Outcomes, Mediators and Methodological Considerations

Supplementary Materials

Supplementary Methods: Detail for Multiple Imputation Methods

Missing data was addressed using the method of Multiple Imputation by Chained Equations (MICE; Azur et al., 2011). Specifically, fifty datasets were imputed, with the imputation regression equation for each variable consisting of (a) all analysis variables with complete data, (b) all analysis variables from the baseline assessment, and (c) all timepoints of the variable being imputed. Inferential analyses were based on pooled estimates using Rubin's rules (Rubin, 1987).

Supplementary Results: Secondary Outcomes

Abstainers were removed in order to examine the secondary outcomes in those who were reducers in smoking. Abstainers were defined as no cigarettes consumed between measurement points) from smoking ($n_{intervention}$ =5; n_{control} = 1) at any time from baseline to 3-months post-intervention before repeating secondary outcomes analyses. The final size of the sample in this analysis was 101 ($n_{\text{intervention}}$ = 49; n_{control} = 52). Tables S1 and S2 present the between and within-groups pairwise comparisons respectively. Both tables present data for samples including and excluding participants who were deemed abstinent at any of the follow-up periods, with within-groups data from the main paper repeated for convenient comparisons. Reductions in craving, nicotine dependence, evaluation of smoking stimuli and motivation to quit were still observed with abstainers removed. However, slightly weaker effect sizes were detected for craving, nicotine dependence and evaluation of smoking stimuli in the intervention group compared to analyses using the full dataset.

Supplementary Methodology and Results: Mediation Models

Method

As per our pre-registered hypotheses, we investigated whether evaluation of smoking stimuli or response inhibition (measured using stop signal reaction time (SSRT) from the stop signal task (SST)) mediated differences in smoking (defined as mean number of cigarettes per day) or craving between the intervention and control groups by extending the three timepoint autoregressive mediation model described by MacKinnon (2008) and Cole and Maxwell (2003) into four timepoint models. This involved estimating autoregressive and cross lagged paths between the outcome (e.g., craving) and the mediator (e.g. evaluation of smoking stimuli) across the four timepoints, as well as using the grouping variable as a predictor of time 1 (post-intervention) and time 2 (1 month follow-up) outcomes. As discussed in MacKinnon (2008) and Cole and Maxwell (2003), the potential mediational paths examined were a1b1 and a2b2 (assuming stationarity of effects), as well as a1b2 and a2b3 (i.e., longitudinal mediation).

Results

Firstly, changes in evaluation of smoking stimuli did not significantly mediate (paths a1b1, a1b2, a2b2 and a2b3) differences in smoking (See Figure S1) or craving for cigarettes (See Figure 2) between groups at follow-ups. In addition, changes in response inhibition did not significantly mediate (paths a1b1, a1b2, and a1b3) differences in smoking (See Figure S3) or craving for cigarettes (See Figure S4) between groups at follow-ups. However, we found that

the cross-lagged relationship of evaluation of smoking images at T2 for predicting smoking (Fig S1) and craving (Fig S2) at T3 were significant. Age was not a significant predictor in these models, except for predicting smoking (Fig 1 and Fig 3) and craving at T2 when controlling for evaluation of smoking images (Fig 2).

Supplementary Methods and Results: Exploratory Independent Effects and Moderators

Method

We conducted exploratory analyses where we investigated whether instead of acting as mediators, the change in response inhibition or devaluation of smoking stimuli acted independent of group, or a moderator of the relationship between group and outcomes (i.e., smoking and craving). In addition, we explored whether age acted as a moderator between condition and specific outcomes: nicotine dependence, craving or devaluation of smoking stimuli. After initial analyses, motivation to quit was also explored as a moderator of reductions in smoking, craving or nicotine dependence. These exploratory independent effects and moderation analyses were conducted in the interest of determining for whom ICT might work best. To perform these analyses, we first regressed the post training assessment of response inhibition, devaluation or motivation on to the pre training timepoint and saved the residuals from these models as a measure of residualised change. A negative residualised change score indicates the participant has improved in their response inhibition or devaluation over time, or that their motivation had decreased. We then included the residualised change scores as predictors in the previously estimated multi-level mixed-effects linear regression models in Stata 15 with a group x residualised change score x timepoint effect providing evidence that the effect of group on the outcome over time was moderated by the amount of change in the moderator over the training period; a residualised change score x timepoint effect was indicative of a change in evaluation of smoking stumuli or response inhibition or motivation to quit independently predicting outcome (i.e. smoking or craving, or also nicotine dependence by motivation). Given the impact of age in the previous paper (Bos et al., 2019) age as a moderator was investigated using mixed effects regression models with group x age x timepoint effect on outcome (i.e. nicotine dependence, craving or devaluation of smoking stimuli).

Results

Residualised change in evaluation of smoking stimuli during the training period independently predicted smoking, B = 2.33, SE = .67, p <.001, [1.02, 3.63]; and craving, B = 9.80, SE = 1.98, p <.001, [5.91, 13.70]; and significantly interacted with time when predicting smoking, F(3, 1510.6) = 4.46, p = .004 (See Table S3 and Figure S5), and craving F(3, 1046.3) = 5.56, p <.001 (See Table S4 and S6). However, there was no significant three-way interaction between group, time and evaluation of smoking stimuli when predicting smoking, F(3, 1510.9) = 1.24, p = .29, or craving, F(3, 1049.6) = .73, p = .53. There were no significant independent or interaction effects of residualised change in response inhibition during the training period on smoking or craving. Additionally, there were no significant independent or interaction effects of age on observed reductions in craving, nicotine dependence or evaluation of smoking stimuli. Independent and interaction effects of the change in motivation to quit on improvements in smoking, craving and nicotine dependence were also non-significant.

References

Azur, M.J., Stuart, E.A., Frangakis, C., and Leaf, P.J. (2011). Multiple imputation by chained equations: what is it and how does it work? Int J Methods Psychiatr Res. 20:1**,** 40-49. doi: 10.1002/mpr.329.

Bos, J., Staiger, P.K., Hayden, M.J., Hughes, L.K., Youssef, G., and Lawrence, N.S. (2019). A randomized controlled trial of inhibitory control training for smoking cessation and reduction. J Consult Clin Psychol 87:9**,** 831-843. doi: 10.1037/ccp0000424.

Cole, D.A., and Maxwell, S.E. (2003). Testing mediational models with longitudinal data: questions and tips in the use of structural equation modeling. J Abnorm Psychol 112:4**,** 558-577. doi: 10.1037/0021-843X.112.4.558.

MacKinnon, D.P. (2008). "Longitudinal Mediation Models," in Introduction to Statistical Mediation Analysis. (New York, NY, USA: Taylor & Francis Group), 193-236.

Rubin, D.B. (1987). Multiple imputation for nonresponse in surveys. New York: Wiley.

Table S1

*Cohen's d effects sizes and 95% Confidence Intervals for Between Groups Comparisons on Variables at Different Timepoints**

	**Baseline (0)**	**Post-Intervention (P)**	**1-Month Follow-Up (1M)**	**3-Month Follow-Up (3M)**
	.06	-.01	.12	-.14
Craving	[-.32, .44]	[-.38, .37]	[-.26, .50]	[-.52, .24]
	.09	-.05	.08	-.19
Non-Abstainers	[-.30, .48]	[-.44, .34]	[-.31, .47]	[-.58, .20]
	.14	-.01	.24	.26
FTND	[-.24, .52]	[-.38, .37]	[-.14, .62]	[-.13, .64]
	.14	-.10	.12	.17
Non-Abstainers	[-.25, .53]	[-.48, .30]	[-.27, .51]	[-.22, .56]
	-.09	-.10		
SSRT (SST)	[-.47, .29]	[-.48, .28]	--------	--------
	-.09	-.11		
Non-Abstainers	[-.47, .31]	[-.50, .28]		
	-.01	0		
Go RT (SST)	[-.39, .37]	[-.38, .38]	--------	--------
	-.08	-.03		
Non-Abstainers	[-.47, .31]	[-.42, .36]		
Evaluation of Images:				
	.01	.10	.21	.11
Smoking	[-.37, .39]	[-.28, .48]	[-.17, .59]	[-.27, .49]
	.06	.07	.17	.06
Non-Abstainers	[-.33, .45]	[-.32, .46]	[-.23, .55]	[-.33, .45]
Relaxing	.38	.05	.26	.31
Activities	[-.01, .76]	[-.33, .43]	[-.13, .63]	[-.08, .68]
	.24	-.03	.17	.22
Non-Abstainers	[-.15, .63]	[-.42, .36]	[-.23, .56]	[-.18, .61]
	.12	.10	.15	-.12
Motivation	[-.26, .50]	[-.28, .48]	[-.23, .53]	[-.50, .26]
	.14	.13	.20	-.07
Non-Abstainers	[-.26, .52]	[-.27, .51]	[-.20, .59]	[-.46, .32]

Note. Negative *d* indicates that the intervention group scored higher than the control group. Non-Abstainers = analysis conducted with participants who were abstinent at any follow-up period removed from analysis. FTND = Fagerström Test of Nicotine Dependence, SST = stop signal task, SSRT = stop signal reaction time (measure of inhibitory control), GO RT = reaction time on go trials.

*All $p > .05$.

Table S2

Descriptive Statistics and Pairwise Comparisons of Each Secondary Outcome by Group over Time with Full Dataset and Non-Abstainers

	Group x Time Interaction on Outcome	Baseline	Post-Intervention		1-Month Follow-Up		3-Month Follow-Up	
		EMM (SE)	EMM (SE)	d_z [95% CI]	EMM (SE)	d_z [95% CI]	EMM (SE)	d_z [95% CI]
Craving	$F(3, 1440.5) = .58, p = .63$							
Intervention		46.44 (3.71)	32.03 (3.97)	-.44** [-.72, -.16]	32.03 (4.04)	-.45** [-.73, -.17]	34.54 (4.71)	-.31* [-.58, -.03]
Control		47.99 (3.68)	31.87 (4.24)	-.48** [-.76, -.19]	35.67 (4.39)	-.35* [-.63, -.08]	29.71 (5.00)	-.47** [-.75, -.18]
Non-Abstainers	$F(3,1360.0) = .75, p = .53$							
Intervention		46.03 (3.92)	33.68 (4.07)	-.38** [-.67, -.09]	33.50 (4.17)	-.39** [-.68, -.10]	36.85 (5.10)	-.23 [-.51, .06]
Control		48.55 (3.72)	32.31 (4.29)	-.48** [-.77, -.19]	35.89 (4.45)	-.36** [-.64, -.08]	30.28 (5.06)	-.47** [-.75, -.18]
FTND	$F(3, 1392.6) = .98, p = .40$							
Intervention		5.41 (.30)	3.80 (.32)	-.78*** [-1.08, -.47]	3.94 (.35)	-.64*** [-.93, -.34]	3.75 (.35)	-.72*** [-1.02, -.42]
Control		5.72 (.31)	3.79 (.33)	-.91*** [-1.22, -.58]	4.57 (.35)	-.50*** [-.78, -.21]	4.43 (.40)	-.48** [-.76, -.19]
Non-Abstainers	$F(3,1227.3) = .79, p = .50$							
Intervention		5.42 (.31)	4.01 (.32)	-.71*** [-1.02, -.39]	4.27 (.36)	-.51*** [-.80, -.21]	4.08 (.36)	-.58*** [-.89, -.28]
Control		5.72 (.30)	3.79 (.33)	-.92*** [-1.25, -.60]	4.58 (.35)	-.50*** [-.78, -.21]	4.52 (.40)	-.45** [-.73, -.16]
SST								
SSRT	$F(1, 578.0) = .01, p = .92$							
Intervention		264.70 (8.82)	264.72 (10.64)	0 [-.27, .27]	--------		--------	

	Group x Time Interaction on Outcome	Baseline EMM (SE)	Post-Intervention EMM (SE)	Post-Intervention d_z [95% CI]	1-Month Follow-Up EMM (SE)	1-Month Follow-Up d_z [95% CI]	3-Month Follow-Up EMM (SE)	3-Month Follow-Up d_z [95% CI]
Control		258.61 (9.33)	256.84 (12.66)	-.02 [-.29, .25]	--------		--------	
Non-Abstainers	*F*(1, 745.2) = .02, *p* = .90							
Intervention		265.97 (9.25)	265.72 (10.62)	0 [-.28, .28]	--------		--------	
Control		260.27 (9.48)	257.75 (12.72)	-.02 [-.30, .25]	--------		--------	
Go RT	*F*(1, 452.1) = <.01, *p* = .97							
Intervention		662.68 (13.63)	652.52 (19.25)	-.07 [-.33, .20]	--------		--------	
Control		661.82 (13.82)	652.52 (16.69)	-.07 [-.34, .20]	--------		--------	
Non-Abstainers	*F*(1, 504.9) = .03, *p* = .86							
Intervention		669.45 (14.08)	656.61 (18.51)	-.09 [-.37, .19]	--------		--------	
Control		661.87 (13.95)	653.50 (16.70)	-.06 [-.34, .21]	--------		--------	
Evaluation of Images								
Smoking	*F*(3, 1638.0) = .30, *p* = .83							
Intervention		51.53 (3.26)	34.32 (3.74)	-.60*** [-.89, -.31]	36.20 (3.75)	-.53*** [-.82, -.25]	33.83 (3.86)	-.60*** [-.89, -.31]
Control		51.76 (3.30)	37.15 (3.86)	-.50*** [-.78, -.21]	41.89 (3.99)	-.33* [-.60, -.05]	36.97 (4.05)	-.48*** [-.77, -.20]
Non-Abstainers	*F*(3, 1776.5) = .12, *p* = .95							
Intervention		50.80 (3.40)	35.81 (3.84)	-.54*** [-.84, -.24]	37.87 (3.79)	-.47** [-.76, -.17]	35.86 (4.09)	-.51*** [-.80 -.21]

	Group x Time Interaction on Outcome	Baseline	Post-Intervention		1-Month Follow-Up		3-Month Follow-Up	
		EMM (SE)	EMM (SE)	d_z [95% CI]	EMM (SE)	d_z [95% CI]	EMM (SE)	d_z [95% CI]
Control		52.19 (3.30)	37.61 (3.85)	-.51*** [-.79, -.22]	42.30 (4.01)	-.33* [-.61, -.05]	37.64 (4.07)	-.48** [-.77, -.19]
Relaxing Activities	*F*(3, 2435.6) = .98, *p* = .40							
Intervention		72.68 (2.31)	75.43 (2.56)	.16 [-.11, .42]	73.21 (2.61)	.03 [-.24, .30]	72.84 (2.50)	.01 [-.26, .28]
Control		79.04 (2.33)	76.34 (2.64)	-.15 [-.42, .12]	78.15 (2.80)	-.05 [-.32, .22]	78.50 (2.61)	-.03 [-.30, .24]
Non-Abstainers	*F*(3, 2936.0) = .71, *p* = .55							
Intervention		74.86 (2.32)	76.46 (2.60)	.09 [-.19, .37]	74.65 (2.60)	-.01 [-.29, .27]	74.13 (2.53)	-.04 [-.32, .24]
Control		78.72 (2.25)	75.98 (2.54)	-.16 [-.43, .12]	77.71 (2.75)	-.05 [-.32, .22]	78.02 (2.55)	-.04 [-.31, .23]
Motivation	*F*(3,1196.5) = .72, *p* = .54							
Intervention		79.25 (3.93)	70.77 (4.29)	-.26 [-.53, .01]	65.04 (4.58)	-.42** [-.69, -.14]	61.29 (5.26)	-.46** [-.74, -.18]
Control		82.80 (3.97)	74.03 (4.37)	$-.27^{*a}$ [-.54, .01]	70.12 (4.60)	-.37** [-.65, -.09]	56.94 (5.20)	-.67*** [-.97, -.37]
Non-Abstainers	*F*(3, 1280.0) = .59, *p* = .62							
Intervention		78.48 (4.13)	70.00 (4.43)	-.27 [-.55, .02]	62.92 (4.72)	-.46** [-.75, -.17]	58.72 (5.56)	-.50** [-.80, -.20]
Control		82.37 (4.01)	73.85 (4.38)	-.26 [-.54, .01]	69.45 (4.66)	-.38** [-.66, -.09]	56.01 (5.26)	-.68*** [-.98, -.38]

Note. The presented within groups d_z is that specific time point compared to baseline. Negative d_z and smaller EMM than baseline denote an improvement in secondary outcomes. EMM = Estimated Marginal Means; SE = Standard Error; CI = confidence interval. Non-Abstainers = analysis conducted with participants who were abstinent at any follow-up period removed from analysis. FTND = Fagerström Test of Nicotine Dependence, SST = stop signal task, SSRT = stop signal reaction time (measure of inhibitory control), Go RT = reaction time on go trials.
$^{*}p < .05$; $^{**}p < .01$; $^{***}p <. 001$. $^{*a}p = .049$.

Figure S1

Mediation of differences in smoking by changes in evaluation of smoking stimuli between groups

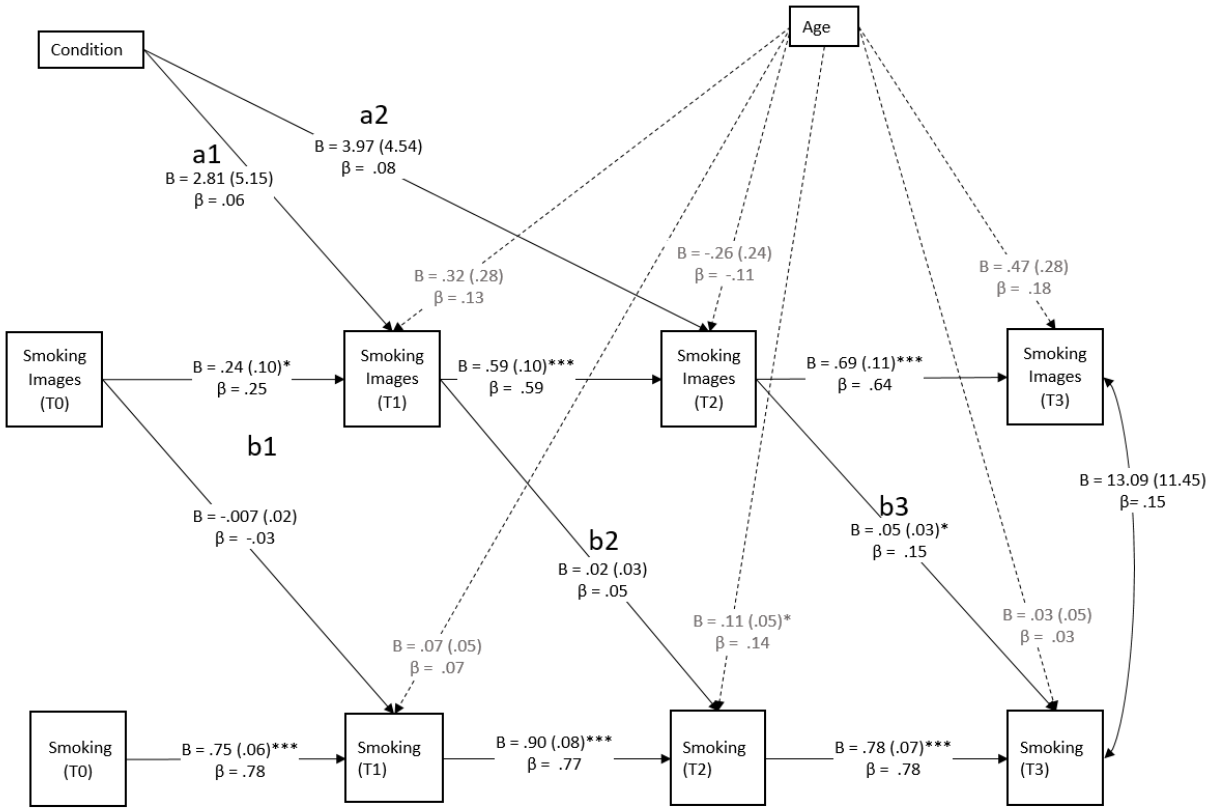

Note. The mediation model shows the cross-lagged relationships between variables: group, evaluation of smoking stimuli (Smoking Images) and smoking. Mediation paths of interest are a1b1 (B = -.02 (.08), p = .78), a1b2 (B = .06 (.15), p = .69), a2b2 (B = .05 (.14), p = .71), and a2b3 (B = .19 (.24), p = .44). Arrows with grey dashed lines represent age-related effects. Coefficients presented are unstandardized beta (B) with standard errors in brackets, and standardised beta (β) as a measure of effect size. T0 = baseline, T1 = post-intervention, T2 = 1-month follow-up, T3 = 3-month follow-up.

*p < .05; **p < .01; ***p < .001.

Figure S2

Mediation of differences in craving by changes in evaluation of smoking stimuli between groups

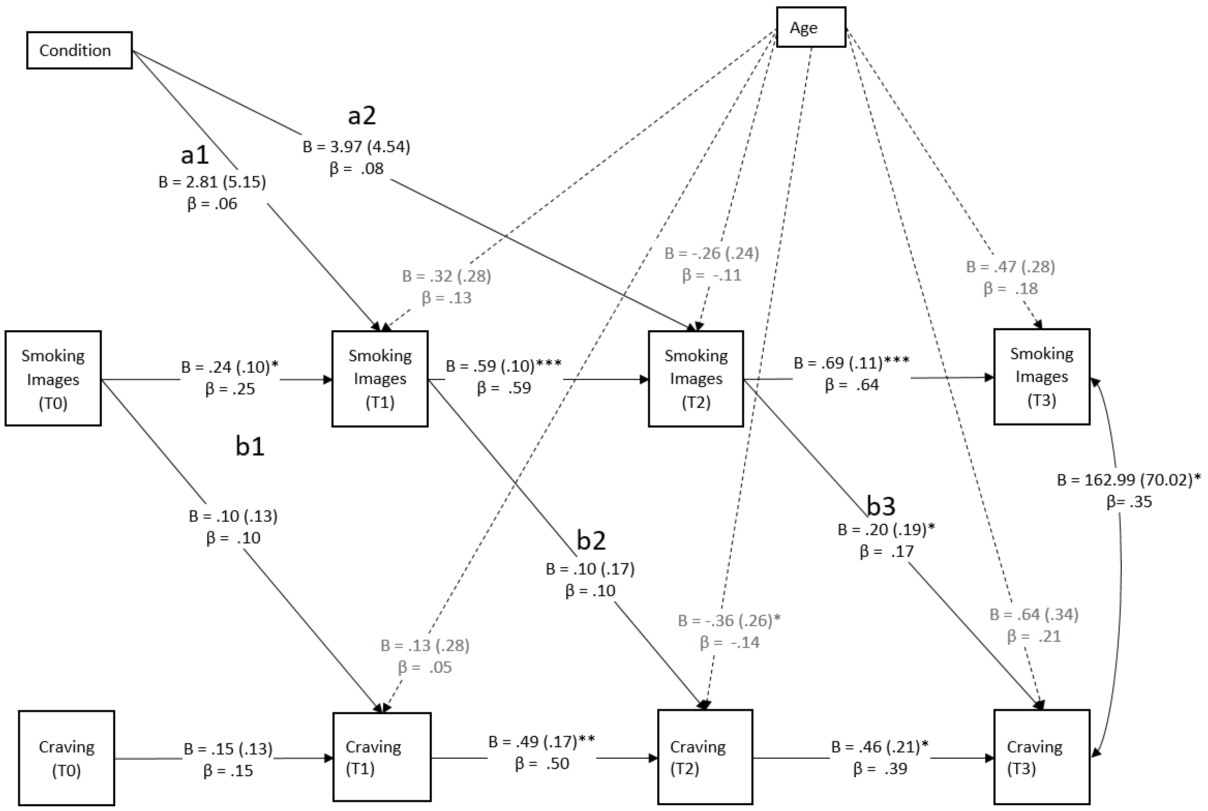

Note. The mediation model shows the cross-lagged relationships between variables: group, evaluation of smoking stimuli (Smoking Images) and craving. Mediation paths of interest are a1b1 (B = .28 (.78), p = .72), a1b2 (B = .38 (1.01), p = .71), a2b2 (B = .36 (.97), p = .71), and a2b3 (B = .86 (1.44), p = .55). Arrows with grey dashed lines represent age-related effects. Coefficients presented are unstandardized beta (B) with standard errors in brackets, and standardised beta (β) as a measure of effect size. T0 = baseline, T1 = post-intervention, T2 = 1-month follow-up, T3 = 3-month follow-up.

*p < .05; **p < .01; ***p < .001.

Figure S3

Mediation of differences in smoking by changes in inhibitory control between groups

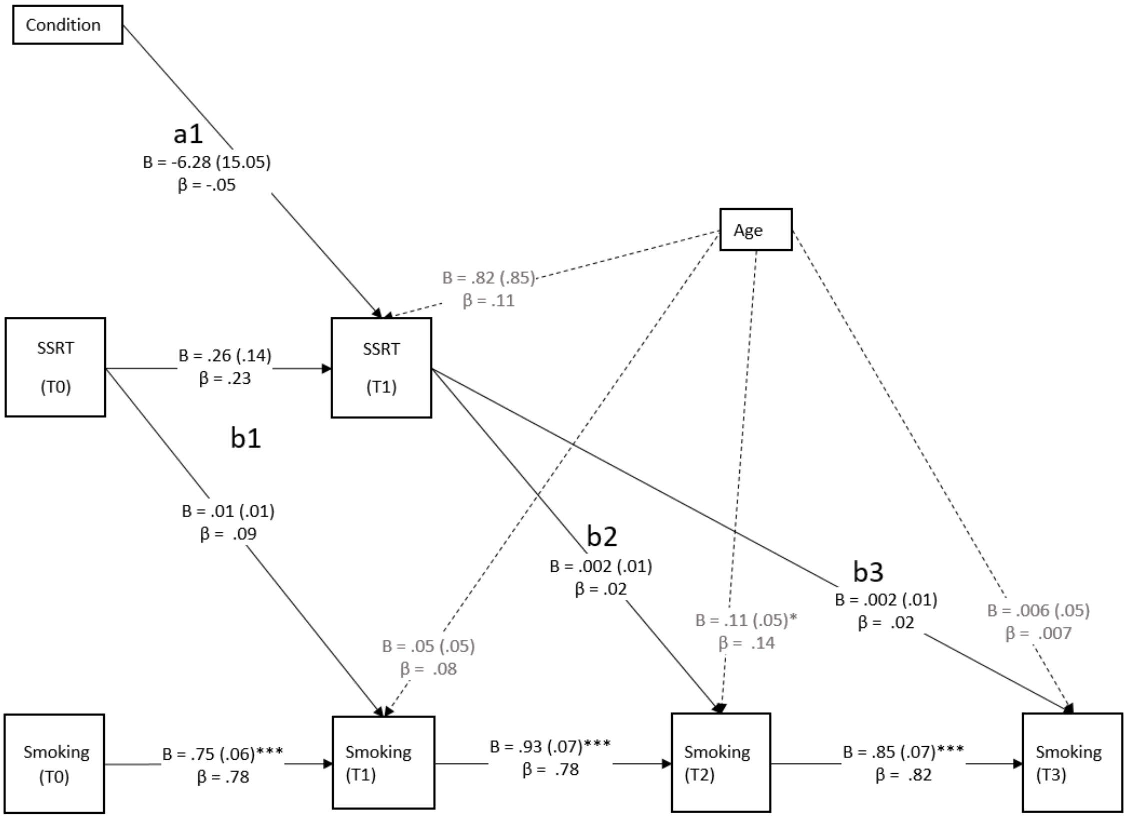

Note. The mediation model shows the cross-lagged relationships between variables: group, inhibitory control and smoking. Mediation paths of interest are a1b1 (B = -.06 (.19), p = .74), a1b2 (B = .01 (.11), p = .93), and a1b3 (B = -.03 (.13), p = .84). Arrows with grey dashed lines represent age-related effects. Coefficients presented are unstandardized beta (B) with standard errors in brackets, and standardised beta (β) as a measure of effect size. T0 = baseline, T1 = post-intervention, T2 = 1-month follow-up, T3 = 3-month follow-up.
*p < .05; **p < .01; ***p < .001.

Figure S4

Mediation of differences in craving by changes in inhibitory control between groups

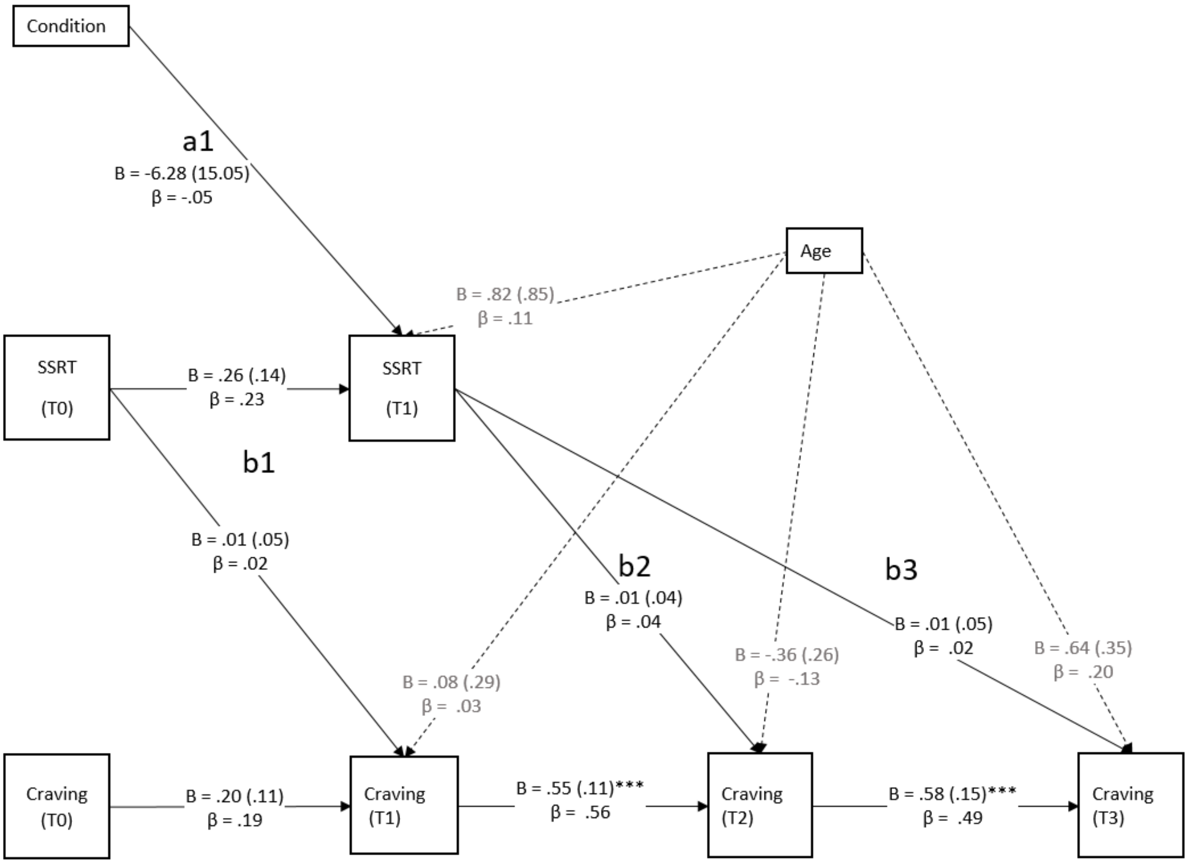

Note. The mediation model shows the cross-lagged relationships between variables: group, response inhibition and craving. Mediation paths of interest are a1b1 (B = -.04 (.63), p = .95), a1b2 (B = -.01 (.64), p = .99), and a1b3 (B = -.13 (.68), p = .85). Arrows with grey dashed lines represent age-related effects. Coefficients presented are unstandardized beta (B) with standard errors in brackets, and standardised beta (β) as a measure of effect size. T0 = baseline, T1 = post-intervention, T2 = 1-month follow-up, T3 = 3-month follow-up. *p < .05; **p < .01; ***p < .001.

Table S3

Rate of change (β) and Standard Error (SE) in evaluation of smoking stimuli for predicting smoking at each time point, independent of group

	β	SE	*p*	95% CI
Baseline	.97	.74	.19	[-.47, 2.41]
Post-Intervention	2.43	.74	.001	[.98, 3.88]
1-Month Follow-Up	2.86	.80	<.001	[1.30, 4.43]
3-Month Follow-up	3.04	.80	<.001	[1.46, 4.62]

Figure S5

Predictive Margins: Smoking by Residuals of Change in Evaluation of Smoking Stimuli- with 95% CIs

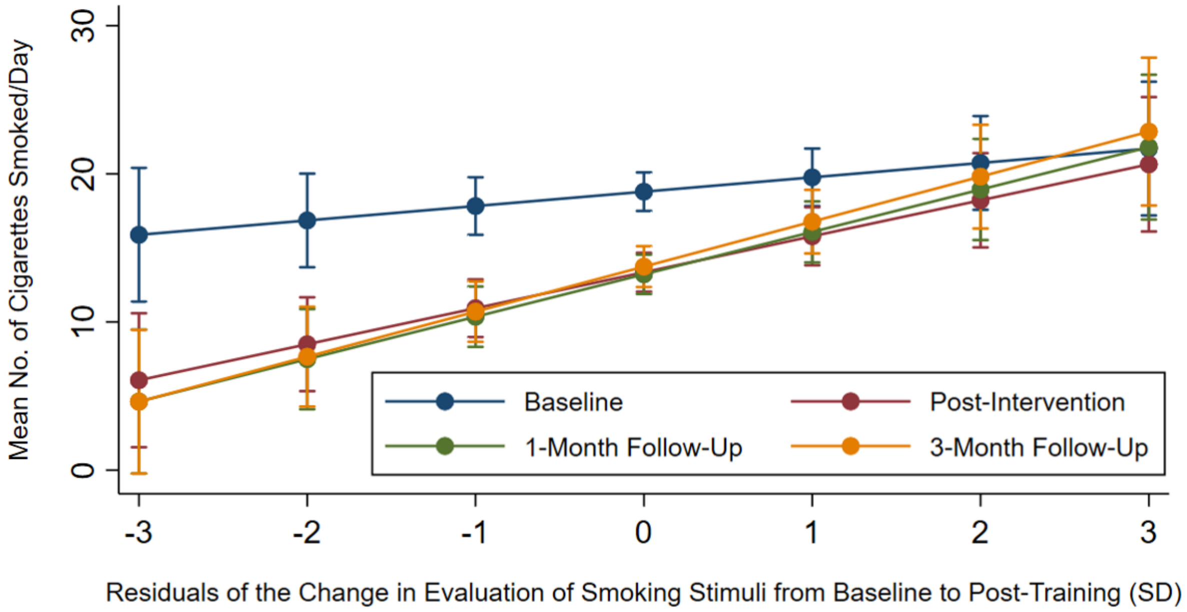

Note. The mean number of cigarettes smoked per day at each time point are shown for each residualised change in evaluation of smoking stimuli between baseline and post-intervention, independent of group. Error bars represent 95% confidence intervals. Negative values are indicative of a devaluation of smoking stimuli. SD = standard deviation.

Table S4

Rate of change (β) and Standard Error (SE) in evaluation of smoking stimuli for predicting craving at each time point, independent of group

	β	SE	*p*	95% CI
Baseline	2.74	2.77	.32	[-2.69, 8.17]
Post-Intervention	15.99	2.78	<.001	[10.53, 21.44]
1-Month Follow-Up	8.44	2.90	.004	[2.74, 14.14]
3-Month Follow-up	12.05	3.01	<.001	[6.13, 17.98]

Figure S6

Predictive Margins: Craving by Residuals of Change in Evaluation of Smoking Stimuli- with 95% Cis

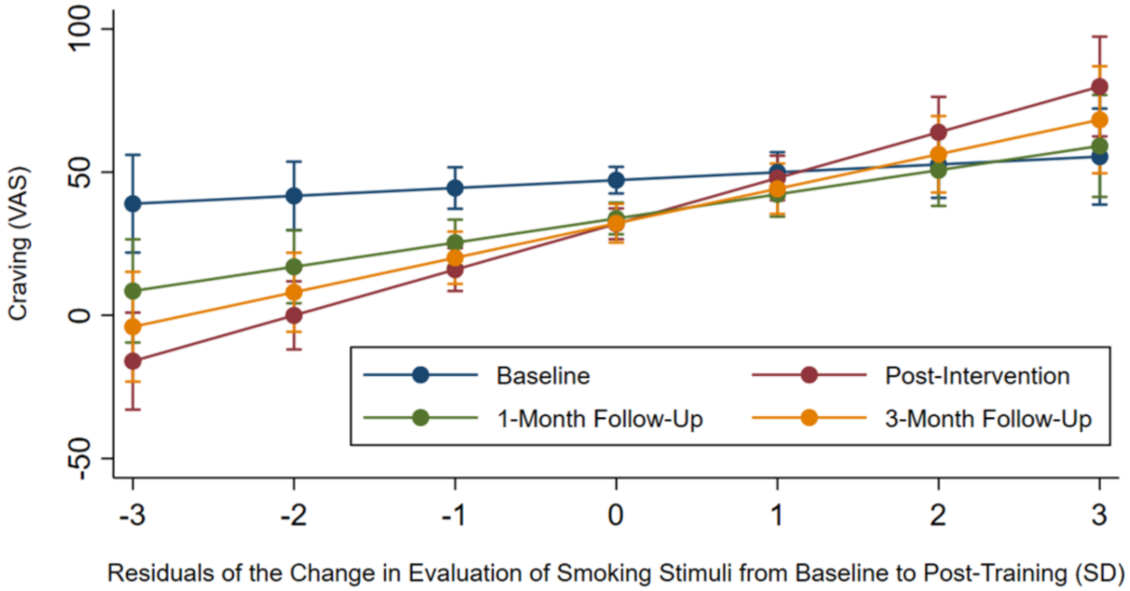

Note. The mean craving scores at each time point are shown for each residualised change in evaluation of smoking stimuli between baseline and post-intervention, independent of group. Error bars represent 95% confidence intervals. Negative values are indicative of a devaluation of smoking stimuli. SD = standard deviation.
